# Substrate-Induced Strain Effect on Structural and Magnetic Properties of La_0.5_Sr_0.5_CoO_3_ Films

**DOI:** 10.3390/nano11030781

**Published:** 2021-03-18

**Authors:** Miriam Sánchez-Pérez, Juan Pedro Andrés, Juan Antonio González, Ricardo López Antón, Marco Antonio López de la Torre, Oscar Juan Dura

**Affiliations:** 1 Department of Applied Physics, University of Castilla-La Mancha, 13071 Ciudad Real, Spain; miriamsanpe13@gmail.com (M.S.-P.); JuanPedro.Andres@uclm.es (J.P.A.); j.a.gonzalez@uclm.es (J.A.G.); Ricardo.lopez@uclm.es (R.L.A.); Marcoantonio.lopez@uclm.es (M.A.L.d.l.T.); 2 Instituto Regional de Investigación Científica Aplicada (IRICA), University of Castilla-La Mancha, 13071 Ciudad Real, Spain; 3 Instituto de Investigaciones Energéticas y Aplicaciones Industriales (INEI), University of Castilla-La Mancha, 13071 Ciudad Real, Spain

**Keywords:** thin films, ferromagnetism, oxides

## Abstract

We present a detailed study about the substrate-induced strain and thickness effects on the structure and magnetic properties of La_0.5_Sr_0.5_CoO_3_ films. The in-plane tensile or compressive strain imposed by four different substrates configures an in-plane or out-of-plane easy axis, respectively. The presence of a soft magnetic phase at the interface is also conditioned by the type of strain. The obtained results are discussed in terms of the different anisotropies that participate and control the final magnetic behavior. The relevance of these results lies in the feasibility of La_0.5_Sr_0.5_CoO_3_ in memory applications and spintronic devices.

## 1. Introduction

Perovskite complex oxides have received great attention in the last decades because of their potential implementation in diverse technological applications as ferromagnetic electrodes, gas separation systems, electrochemical reactors, multiferroic and thermoelectric applications, or spintronic devices [1,2]. Among them, strontium doped lanthanum cobaltite La_1−x_Sr_x_CoO_3_ (LSCO) is one of the most studied complex oxides due to its intriguing fundamental behavior. The first works about the fundamental properties of this system were performed in the 1950s by G. Jonker and J. Van Santen [3], and J. Goodenough [4]. Without strontium doping, the lanthanum cobaltite LaCoO_3_ is an insulating nonmagnetic compound with a rhombohedral structure [5]. In this case, a magnetic transition was observed around 50–90 K, in addition to a metal–insulator transition around 500–600 K [6,7]. These transitions can be understood in terms of spin transitions, whose nature is still debated [7,8,9,10,11,12,13]. These spin transitions can be also reached by strontium-induced hole doping, stabilizing the spin states of the system. The substitution of La^3+^ by Sr^2+^ induces the oxidation of the CoO_3_ matrix, giving rise to a semiconducting behavior of the La_1−x_Sr_x_CoO_3_ compound [14,15,16]. Its phase diagram is still being studied [5,14,17,18,19,20], although the current consensus indicates that a doping of x = 0.18 is the critical point for a metal–insulator transition [17]. Under light doping conditions (x < 0.18), magnetoelectronic phase separation (MEPS) has been proposed to unveil its complex phase diagram. MEPS is defined as the spatial coexistence of multiple electronic and magnetic phases without chemical segregation [16,17,21,22,23,24]. Hence, there is a coexistence of ferromagnetic clusters embedded on a nonmagnetic matrix, following the cluster-glass ferromagnetic model. When the hole doping increases, the cluster density is increased, leading to a percolation transition to a metallic and long-range ferromagnetic order state above the critical point (x = 0.18). For high strontium doping (x > 0.18), the Curie temperature increases, reaching a maximum of around 240 K at x = 0.5, which corresponds to LSCO with a pseudocubic structure (a = 3.84 Å) [17,20,21]. This maximum in T_c_ can be interpreted in terms of the 1:1 ratio of Co^3+^:Co^4+^, maximizing the double-exchange interaction [17]. A clear understanding of these changes in the electric and magnetic properties as a function of Sr doping is still under consideration [22,25,26]. While several authors indicated that double-exchange interaction (Co^3+^-Co^4+^) could be the responsible [18,27], other works present the competition of superexchange (Co^3+^-Co^3+^, Co^4+^-Co^4+^) and double-exchange interactions as the reason of these phenomena [28,29]. 

The study of this compound in thin film form has also received remarkable attention. Firstly, the confinement of the magnetoelectronic phase separation, or its emergence in thin film samples, can lead to changes in the properties of the compound, e.g., increasing strongly the coercivity with potential applications in permanent magnets [16,26]. It is also of interest to study the influence of the film thickness on the magnetic properties of ultrathin films, with relevant applications in data storage devices [30]. The role of the substrate-induced strain and its relaxation in the perpendicular direction to the sample when the film thickness increases is to provide tunability to the properties of the compound. Hence, strain engineering can be a potential tool to modify the state and properties of a compound as, for example, the magnetic properties. An excellent example of the power of the strain control can be the insulating nonmagnetic LaCoO_3_ compound, which can present strain-stabilized ferromagnetic behavior in thin film form [31,32]. Another example showing the importance of strain control is the possibility to modify or tune the magnetic anisotropies in the sample. Special attention should be given to the magnetocrystalline and magnetoelastic anisotropies induced by the substrate strain. Different effects of substrate strain have been reported recently, such as the change of the easy axis as a result of the strain [33,34] or the strong dependence of the coercivity on the magnetocrystalline anisotropy controlled via strain [35,36,37]. 

In this work, we present an analysis of the substrate-induced strain influence on the structural and magnetic properties of the perovskite complex oxide LSCO films. We focus on x = 0.5 LSCO, which displays a cubic structure (lattice parameter, a = 3.84 Å) and ferromagnetic behavior. For this, we grow LSCO films on four different substrates that impose both in-plane tensile and compressive strains by means of its different lattice in-plane parameters, varying from 3.75 Å to 3.90 Å. The effect of the relaxation in the direction perpendicular to the sample is concomitantly considered by means of the growth of LSCO films with different thicknesses ranging between 7 and 40 nm. X-ray reflectivity and diffraction as well as magnetometry measurements were performed to characterize the structural and magnetic properties of the films. The measurements show a dependence of the magnetic easy axis with the type of strain imposed and point to the competition between magnetoelastic and magnetocrystalline anisotropies as the main source. The ability to stabilize the easy axis and understanding its mechanism results in great interest, both fundamental and applied. These results could pave the way for advanced applications, for example, in magnetic and magneto-optical storage media [38,39]; fuel cells batteries, electrochemical, and water splitting processes [40]; or in the hot pursuit of spintronics devices [41].

## 2. Experimental Section

LSCO films were deposited by reactive magnetron sputtering from La_0.5_Sr_0.5_CoO_3_ target compound. The LSCO target was prepared by solid-state reaction from SrCO_3_, Co_3_O_4_, and La_2_O_3_ starting powders. These powders were mixed and cold pressed in a 2-inch diameter disk and sintered at 1100 °C and 1200 °C for 48 h and 24 h, respectively [17,21,22,42]. X-ray diffraction, energy-dispersive X-ray spectroscopy, and magnetometry measurements were performed to check the stoichiometry and ferromagnetic behavior of the target material. Commercial single crystal substrates were obtained from Mateck GmbH. Four different substrates were used while attending to the lattice parameter to impose tensile or compressive strain. Therefore, considering that the bulk (i.e., relaxed) lattice parameter of the LSCO is 3.84 Å, we selected SrTiO_3_ (STO; a = 3.90 Å) and Sr_0.3_La_0.7_Al_0.65_Ta_0.35_O_3_ (LSAT; a = 3.87 Å) for applying the tensile in-plane strain and LaAlO_3_ (LAO; a = 3.82 Å) and Sr_0.3_La_0.7_AlO_4_ (SLAO; a = 3.75 Å) for applying the in-plane compressive strain. LSCO films were simultaneously grown on these four substrates with fixed sputtering conditions in order for us to compare the substrate-induced strain effect more accurately. In addition to this, LSCO films with different thicknesses were obtained by varying the deposition time in order to explore the effect of the film relaxation in the growth direction. Hence, four representative thicknesses, i.e., 7 nm, 14 nm, 28 nm, and 40 nm, were obtained. The growth of all LSCO films was on-axis, with the four substrates placed on a heater at a source-to-substrate distance of 11 cm approximately. Prior to deposition, the substrates were heated in a working atmosphere at 800 °C for 10 min. The working atmosphere was a combination of oxygen and argon gases with a total pressure of 9·10^−2^ mbar. The ratio of partial pressures O_2_/Ar and the deposition temperature were fixed to 0.4 and 800 °C, respectively, after several studies to simultaneously obtain the optimal structural and magnetic properties on the four analyzed substrates. The deposition rate was 2 Å/min approximately. During postdeposition, the LSCO films were cooled in the working atmosphere down to 500 °C. At this point, to avoid the formation of oxygen vacancies, the samples were annealed in 700 mbar of oxygen atmosphere at 500 °C for 10 min, and finally cooled in oxygen atmosphere down to room temperature. The final grow conditions were selected after many rounds of trial-and-error while following scanning electron microscopy (SEM), electrical resistivity, and magnetometry properties. The final reproducibility obtained was very high.

X-ray reflectivity (XRR) and diffraction (XRD) measurements were performed with a Cu Kα Philips X’Pert MRD diffractometer by scanning 2θ from 0.4° to 9° and from 20° to 60° respectively (figures only show data in the range of interest for a better comparison). The analysis of the spacing between Kiessig fringes in the XRR scans allowed us to estimate the thickness of the films. Additionally, the crystal domain size in the out-of-plane direction was calculated from the XRD peaks by using the Scherrer relation, once the instrumental broadening was discounted. Magnetometry measurements were performed on a commercial Evercool MPMS-XL Superconducting Quantum Interference Device system (Quantum Design). The magnetization versus temperature (MT) curves were obtained under an applied magnetic field of 100 Oe between 5 and 300 K. All MT curves were measured with the magnetic field direction parallel to the film surface (in-plane). The hysteresis loops (MH) were measured at 5 K under applied magnetic fields between 50 and −50 kOe (again, figures only show data in the range of interest for a better comparison), after field-cooling from room temperature under a magnetic field of 10 kOe. The MH curves were measured in both in-plane and out-of-plane dispositions (i.e., magnetic field direction parallel and perpendicular to the film surface, respectively). Clean substrates were fully characterized to allow for a better correction of its diamagnetic signal, which was considered in the magnetic measurement of the LSCO films.

## 3. Results and Discussion

### 3.1. Structural Characterization

**Figure 1:**
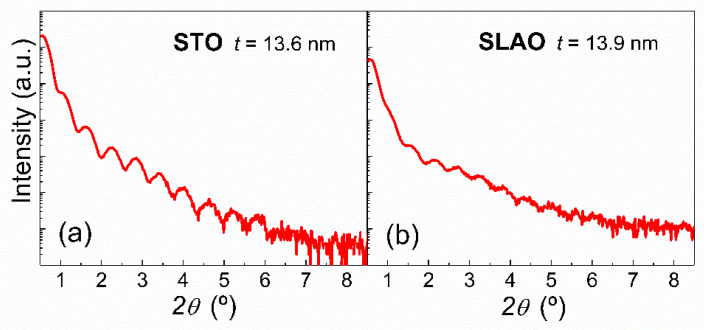
X-ray reflectivity measurements of La_1−x_Sr_x_CoO_3_ (LSCO) films grown on SrTiO_3_ (STO) (**a**) and Sr_0.3_La_0.7_AlO_4_ (SLAO) (**b**) substrates for one representative thickness (about 14 nm).

The X-ray reflectivity (XRR) measurements shown in Figure 1 were performed on LSCO films grown on STO and SLAO substrates, with an approximate thickness of 14 nm. They display well-defined oscillations with slight differences between the analyzed substrates due to the chemical contrast. These measurements allow us to calculate the thickness corresponding to each sample [43,44,45], with an error within the range of 0.1–0.4 nm. The thicknesses calculated for films grown simultaneously on different substrates, i.e., samples with the same deposition time, are very similar, as we expected. Hereafter, the thicknesses will be used to refer to each sample.

**Figure 2:**
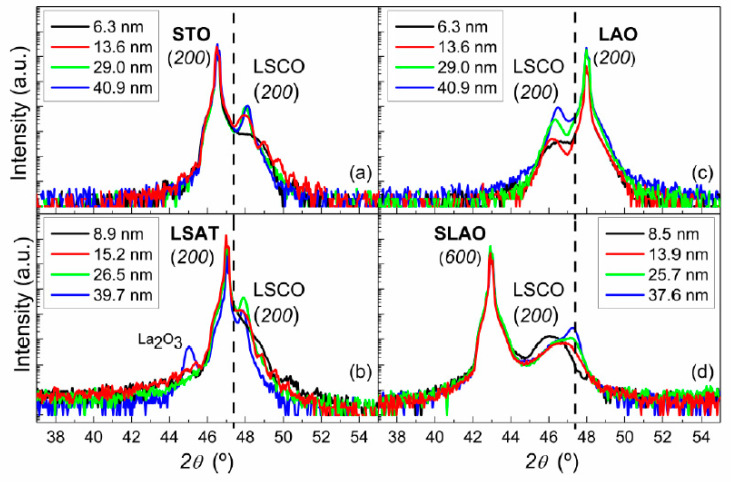
X-ray diffraction scans for LSCO films of various thicknesses grown on STO (**a**), Sr_0.3_La_0.7_Al_0.65_Ta_0.35_O_3_ (LSAT) (**b**), LaAlO_3_ (LAO) (**c**) and SLAO (**d**) substrates. Dashed lines indicate the position of the (200) peak corresponding to LSCO bulk.

X-ray diffraction measurements were performed in θ–2θ geometry on all the samples. Figure 2 shows the region of interest of these scans on all the samples studied. These scans are sensitive to the crystallographic structure in the direction normal to the plane (z). Thus, the LSCO films grown on the in-plane tensile substrates (STO and LSAT substrates) display a lattice parameter smaller than the bulk value, whereas LSCO films grown on in-plane compressive substrates (LAO and SLAO substrates) display larger lattice parameter, as expected.

For LSCO films with thicknesses below 30 nm, the scans performed for the four different substrates show only (h00) reflections, which indicates that samples grow along the z direction, keeping this family of planes (h00). In reference to the thickest LSCO films, the same family of planes is observed for samples grown on STO and LAO substrates. However, in the case of LSAT and SLAO substrates, different peaks (not shown) corresponding to the family (110) were also observed. Additionally, for LSCO films grown on SLAO substrate, a relaxation of the film in the z-direction is evidenced by a splitting and displacement of the peak as the thickness increases, approaching the (200) peak position of LSCO bulk. Although this is also hinted in the sample deposited on the LAO substrate, this behavior is clearly observed only on films grown on SLAO substrate and is most probably due to the largest strain value imposed by the SLAO (2.3%). However, it is noteworthy that for the thinnest film grown on SLAO, the film appears strained. On the other hand, a peak corresponding to La_2_O_3_ oxide was observed only for the thickest film grown on the LSAT substrate. Nevertheless, as it is further discussed, no effect of this compound on the physical properties of the film was found. 

**Figure 3:**
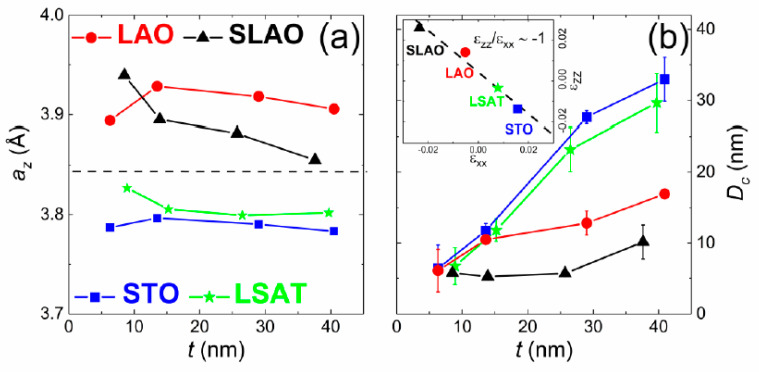
Thickness and substrate-induced strain dependencies of lattice parameter (**a**) and estimated crystal size (**b**) in the z-direction of LSCO films grown on STO, LSAT, LAO, and SLAO substrates. The dashed line in (**a**) indicates the lattice parameter value of LSCO bulk (3.84 Å). Inset shows out-of-plane lattice strain versus in-plane lattice strain, with a linear fit presented by the dashed line (see text).

The out-of-plane pseudocubic lattice parameter (*a_z_*) of each film was calculated from the (200) peak, and their thickness dependence is shown in Figure 3a. For the thinnest LSCO films, there is a clear trend obeying the substrate strain: The LSCO film with the largest lattice parameter *a_z_* corresponds to the lowest in-plane lattice parameter substrate, i.e., the most in-plane compressive substrate (SLAO substrate), whereas the sample with the lowest *a_z_* value matches the largest in-plane lattice parameter substrate, i.e., the most in-plane tensile substrate (STO substrate). This situation would suggest epitaxial growth for the four different substrates, at least for the thinnest films. However, as the film thickness increases, two different behaviors were observed, depending on the compressive or tensile substrate strain. For LSCO films obtained on SLAO and LAO in-plane compressive substrates, there is a generally downward trend of the film lattice parameter as the thickness increases, approaching the bulk lattice parameter value more markedly for the SLAO case. On the other hand, for LSCO films grown on the LSAT and STO in-plane tensile substrates, we observed an approximately constant behavior of the lattice parameter when the film thickness increases, which is 3.80 Å and 3.79 Å, respectively. All this suggests a greater capability of this material to support tensile than compressive strains.

We compared the elastic behavior in the inset of Figure 3b by plotting the out-of-plane lattice strain, Ɛ_zz_ = (*a_z_* − *a_c_*)/*a_c_*, versus the in-plane lattice strain, Ɛ_xx_ = (*a* − *a_c_*)/*a_c_*. Here, *a_c_* stands for the bulk lattice parameter, and *a* is the substrate lattice parameter, assumed as the in-plane lattice parameter for the thinnest LSCO film. Thus, the lineal relation shown as dashed line in the figure confirms the expected Poisson ration ν = 1/3, and it suggests an epitaxial growth of the LSCO films [32]. 

Aside from this, using the full width at half maximum (FWHM) of the (200) peak corresponding to LSCO films, we estimated the crystalline domain size (D_c_) for each film in the out-of-plane direction. The thickness dependence of D_c_ for the group of LSCO films is shown in Figure 3b, indicating again two different trends related to the compressive and tensile substrate strain. While in LSCO films grown on in-plane tensile substrates, the crystal size linearly increases as the thickness does (the crystal grains reaching almost the maximum size allowed by the thickness of the film), those films grown on in-plane compressive substrates behave differently. The increase of D_c_ is fairly gentler in the case of the LAO substrate, while there is almost no increase for the case of the SLAO substrate, indicating that the films grown on the SLAO substrate would remain more relaxed. Hence, the evolution of both the lattice parameter and crystal size suggests that the tensile strain is maintained up to larger thicknesses. It indicates a greater feasibility to support the in-plane tensile strain by LSCO, which agrees with previous studies performed on thin films [46].

**Figure 4:**
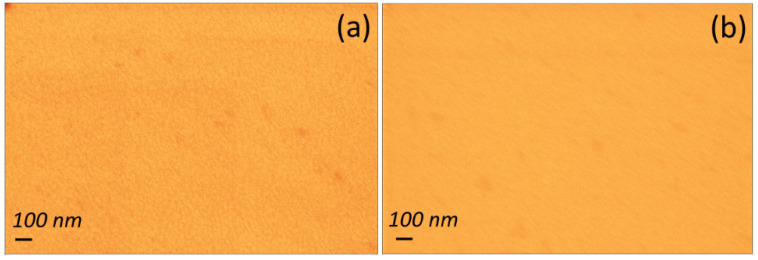
Scanning electron microscope images of LSCO films grown on STO (**a**) and LAO (**b**) substrates for the ~28 nm thickness.

To analyze the surface morphology of the films, SEM images were obtained. In Figure 4 the surface of two samples corresponding to both tensile and compressive cases can be compared. The SEM inspection allowed us to exclude the presence of breaks, defects, or inhomogeneities thorough the samples’ surface, which could influence the subsequent magnetic characterization.

### 3.2. Magnetic Properties

**Figure 5:**
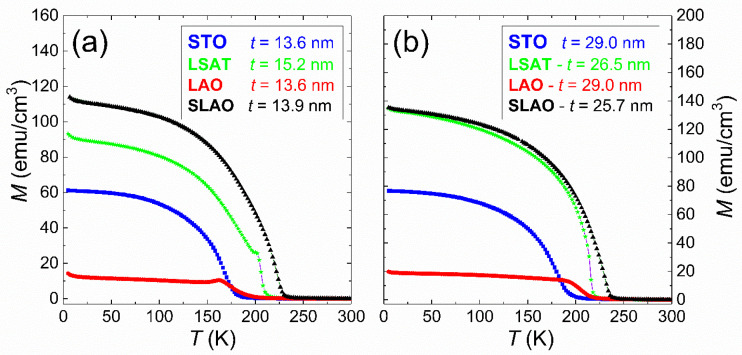
Field-cooled magnetization curves under in-plane magnetic field of 100 Oe performed on LSCO films grown on STO, LSAT, LAO, and SLAO substrates for two representative thicknesses, around 14 nm (**a**) and 28 nm (**b**).

In view of the differences observed in the structural characterization we analyzed its effect on the magnetic behavior. Figure 5 shows the field-cooled (FC) magnetization curves performed on LSCO films grown on STO, LSAT, LAO, and SLAO substrates for two representative thicknesses, around 14 nm (a) and 28 nm (b). These data were taken under an in-plane magnetic field of 100 Oe between 5 and 300 K. The curves obtained evidence of ferromagnetic behavior on all studied films, showing a decrease of the magnetization as we approach the Curie temperature, T_C_. The shoulder observed nearly below T_C_ in some curves, as seen for example, in the film grown on LSAT (15.2 nm), is related with the Hopkinson effect [47,48]. This appears when magnetic anisotropy decreases with the temperature faster than magnetization does, and it was confirmed in these samples when we observed that the feature disappears when the field applied during the measurement increases. We estimated the Curie temperature of each sample as the maximum of the first derivative of the curves, and accordingly we obtained the thickness dependence depicted in Figure 5a. The obtained values for the T_C_ are below the corresponding value to LSCO bulk (around 240 K). Similar findings have been already reported in the literature, and they were mainly related to the finite size effect and/or the oxygen stoichiometry [30,49,50]. However, an ascendant tendency of the T_C_ values as the film thickness increases was observed for the four studied substrates, approaching the LSCO bulk value. Again, a different trend was observed depending on the compressive or tensile substrate strain. For LSCO films grown under tensile strain, on the STO and LSAT substrates, the T_C_ value moderately increases as the thickness does, and it reaches an almost stable value around 30 nm. In the compressive cases, LSCO films grown on the LAO substrate display the largest rise, whereas samples grown on the SLAO substrate show a sharp rise up to 30 nm, decreasing strongly afterwards. This behavior totally agrees with the structural details obtained by XRD, as LSAT is the case of the largest strain and is where more relaxation was seen when the thickness increases. 

**Figure 6:**
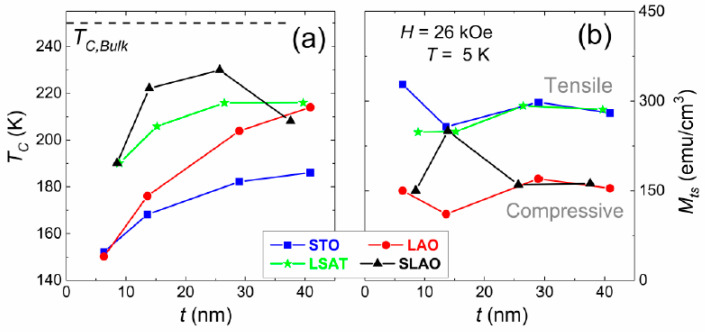
(**a**) Curie temperature (T_C_) dependence on the thickness and the substrate-induced strain; the dashed line at 250 K corresponds to the T_C_ value for LSCO bulk. (**b**) Saturation magnetization (M_ts_) at 5 K for LSCO films grown on STO, LSAT, LAO, and SLAO substrates.

From the hysteresis loops (MH), the magnetization values at 30 kOe were obtained and are plotted in Figure 6b as a function of thickness. The MH curves were measured at 5 K with the magnetic field direction parallel to the surface of the sample (in-plane). The magnetization observed in the LSCO films grown with in-plane tensile strain, i.e., the STO and LSAT substrates, displays values around 300 emu/cm^3^, which are similar to those found in the literature [21,51,52]. However, a different trend was obtained again for the in-plane compressive strain cases, i.e., the SLAO and LAO substrates, displaying values markedly lower.

**Figure 7:**
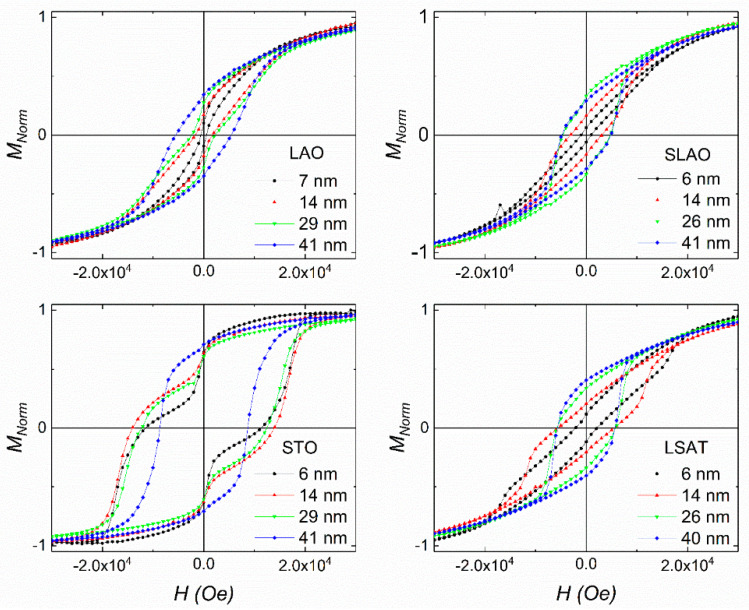
Normalized in-plane hysteresis loops performed at 5 K on LSCO films grown on LAO, SLAO, STO, and LSAT substrates.

The hysteresis loops measured on LSCO films grown are shown in Figure 7 for the four studied substrates. The magnetization values were normalized to the 30 kOe value for a better comparison. Additionally, absolute values of magnetization at 30 kOe (3 T) were compared in Figure 6b for a complete characterization.

Again, the shape of the loops points to a different magnetic behavior of LSCO films, depending on the type of strain imposed by the substrate and the thickness of the film. The LSCO films grown on substrates under in-plane compressive strain (LAO and SLAO) display larger hysteretic behavior, with coercive fields values up to 5 × 10^4^ Oe, as the film thickness increases. In addition, they show a slight wasp-waist shape in the thinner films, more markedly in the case of LAO substrates. The LSCO films grown under tensile in-plane strain display a different trend. On the one hand, loops mostly present larger hysteretic behavior than those obtained for the substrates imposing compressive strain, with coercive fields values up to 1.4 × 10^4^ Oe. On the other hand, the thickness dependence of the hysteresis is not the one observed for the previous case. From the films grown on STO, i.e., the largest tensile strain, two features stand out: The thickest sample shows the lowest hysteresis in comparation with the thinner films, additionally presenting a well-defined step-like shape for field values below 1 × 10^4^ Oe. The step-like feature indicates the presence of two different magnetic phases, i.e., hard and soft, whose relative contributions depend on thickness. This behavior suggests the soft one comes from the interfacial effect between the substrate and the film. Therefore, the relative contribution of the soft one decreases as the total thickness is increased, and thus its presence in the loops is less evident as the film’s thickness increases [53]. Similar results were reported in magnetic thin films, and interesting conclusion were obtained by Rigato et al., which points to an effect associated with the strain acting on surfaces edges [54]. LSCO films grown on a LSAT substrate show a similar behavior but much less marked, pointing to the strain as the main source of the step-like feature. 

**Figure 8:**
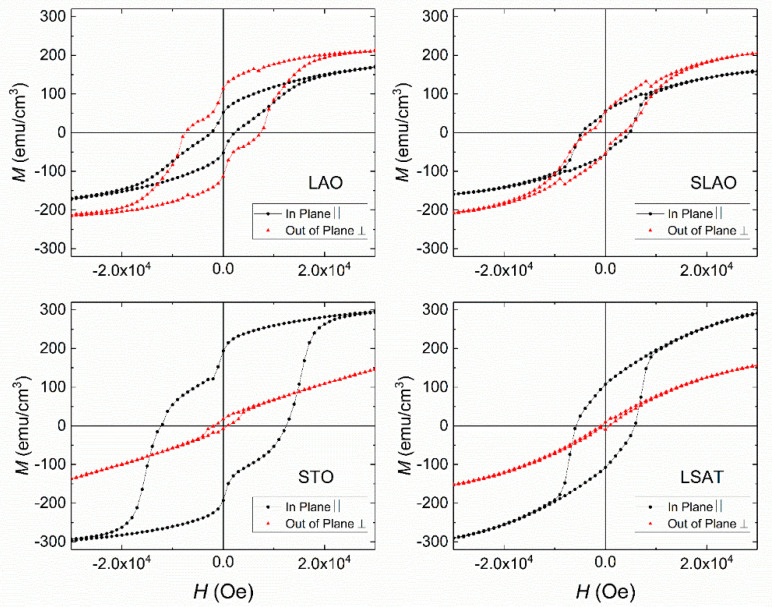
In-plane (IP) and out-of-plane (OOP) hysteresis loops performed at 5 K on LSCO films grown on the LAO, SLAO, STO, and LSAT substrates for the ~28 nm thickness.

In view of the dependence of both structural and magnetic behavior related to the substrate and strain imposed, we will consider the role played by the different anisotropies in the LSCO films. Therefore, different contributions related to shape anisotropy, magnetocrystalline anisotropy, and magnetoelastic anisotropy are participating simultaneously on the films, and they could be modified or induced by the substrate strain [33,34,35,36,37]. In order to elucidate the substrate-induced strain effect and the role played by the anisotropy, hysteresis loops with the magnetic field applied in the direction that is perpendicular to the surface of the sample (out-of-plane) were analyzed. The in-plane tensile or compressive substrate strain must be considered together with the corresponding crystalline elongation or shortening in the out-of-plane direction of the film [30,55]. This crystalline anisotropy, a consequence of the substrate strain, could lead to the change of the magnetic response in each direction. The out-of-plane (OOP) hysteresis loops are compared to the in-plane (IP) curves in Figure 8 for LSCO films with around 28 nm thickness for all the substrates. The OOP measurements display some outliers near 10 kOe because at those fields, the raw moment (before removing the substrate contribution) crosses zero, which makes the values noisier. 

Depending on the type of strain induced by the substrate, two different trends related with the magnetization values and the shape of the loops were observed. Loops for LSCO films grown on STO and LSAT substrates (i.e., the in-plane tensile strain cases) show OOP magnetization values significantly lower than the IP case for the same applied magnetic field, and they show an almost negligible hysteretic behavior. In fact, this is the expected behavior for thin films where the magnetic anisotropy induces an in-plane magnetic easy axis. However, for LSCO films grown on LAO and SLAO (i.e., under in-plane compressive strain), quite similar magnetization values were found for both IP and OOP configurations. Actually, the magnetization in the OOP loops is slightly larger than the one displayed by the IP loops. This behavior, presenting a similar value of both IP and OOP magnetizations, suggests an easy axis of the magnetization not in the plane of the film but somewhere closer to the normal. Such behavior has been previously reported [33,34,35,37]. In particular, Y. Heo et al. [33] found this behavior for LSCO films grown on LSAT and LAO substrates, and they correlated the magnetic anisotropy with the oxygen octahedral distortions in LSCO. In the present work, we confirm experimentally these results in both LSAT and LAO substrates, and we go further by extending the analysis for two more cases: one in-plane tensile substrate (STO substrate) and one in-plane compressive case (SLAO substrate). In summary, the analysis of this set of four substrates suggests that when the strain induced by the substrate is compressive, the magnetization tends to get out of plane to minimize energy, while for the cases of in-plane tensile strain, the magnetization remains in-plane.

With regards to the step-like feature, it presents a particular behavior depending on the substrate strain character. For LSCO films grown on the STO in-plane tensile substrate, the step-like was clearly observed in the IP hysteresis loops, and it is not present in the OOP case. However, for the samples grown on LAO and SLAO in-plane compressive substrates, a well-defined step-like was observed in the OOP magnetization curves. By comparing the obtained results, we deduced that the step-like feature appears when the magnetic field direction is parallel to the elongation of the lattice (the IP loops for the samples grown on STO and LSAT substrates, and the OOP loops for the samples grown on LAO and SLAO substrates). By contrast, when the magnetic field direction is parallel to the shortening of the lattice (the OOP loops for samples grown on STO and LSAT substrates, and the IP loops for samples grown on LAO and SLAO substrates), the hysteresis loop does not show the step-like feature. Therefore, it follows that the source of the step-like signature is related with the lattice distortion imposed by the strain.

**Figure 9:**
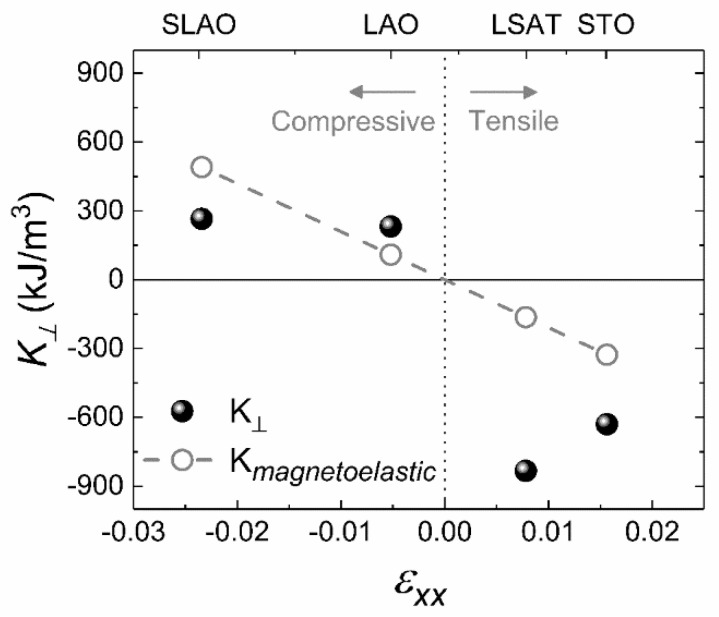
Closed circles correspond to the perpendicular magnetic shape anisotropy (K_⊥_) for LSCO films grown on tensile and compressive substrates, whose thickness is around 28 nm. Open circles and dashed line represent the magnetoelastic anisotropy contribution to K_⊥_ (see text).

The obtained results point to the relevant role of the substrate-induced strain on the magnetic anisotropy for the LSCO thin films. For films presenting an out-of-plane magnetization easy axis, the magnetic shape anisotropy K_S_ must be overcome by other sources of anisotropy. It is convenient to compare the shape anisotropy K_S_ with the perpendicular magnetic anisotropy *K*_⊥_, which includes both magnetocrystalline and magnetoelastic anisotropies. The values of *K_Ʇ_* are obtained from the effective magnetic anisotropy K_eff_, which is calculated from the area enclosed between the OOP and the IP semi-loops. Thus, *K*_⊥_ = K_eff_ − K_S_*,* with $K_{S}=-\mu_{0}\frac{M_{S}^{2}}{2}$, where *M_S_* is the saturation magnetization [56]. *K*_⊥_ values obtained for films with a thickness around 28 nm grown on the four different substrates are compared in Figure 9. The negative *K*_⊥_ values correspond to the LSCO films in which the magnetic shape anisotropy (always negative) dominates, and the magnetization lies in-plane. This situation corresponds to the films grown on the in-plane tensile LSAT and STO substrates. On the other hand, samples displaying positive *K*_⊥_ values correspond to a situation where the magnetic shape anisotropy is surpassed by other anisotropies (this being the case for the samples grown on LAO and SLAO in-plane compressive substrates). From these measurements, both contributions to the perpendicular magnetic anisotropy, i.e., magnetocrystalline and magnetoelastic, cannot be resolved independently. However, a coarse estimation of the magnetoelastic anisotropy using the expression:

(1) $$K_{magnetoelastic}=-\frac{3}{2}\frac{Y}{1+\mu }\lambda_{100}\frac{a_{bulk}-a_{film}}{a_{bulk}}$$

where *λ*_100_, *μ*, and *Y* denote the magnetostriction, the Poisson ratio, and the Young’s modulus, respectively [56], offers blurred results. This estimation points to a contribution caused by both magnetoelastic and magnetocrystalline anisotropies without a clear dominance. Considering these two anisotropies present equivalent symmetry in cubic structures [54], this analysis supports the easy axis obtained in each case. Hence, these results confirm the influence of the strain on the magnetic behavior on LSCO films, which is required for spintronic and advanced memory devices. It should be noted that for the largest in-plane compressive case (SLAO substrate), the results obtained and displayed in Figure 9 are misleading, but the reason could be the large compressive strain we discussed above, which leads to show no trend in structural and magnetic characterization.

## 4. Conclusions

LSCO films were grown with various thicknesses on different tensile and compressive strain substrates to analyze their effect on the structural and magnetic properties of the system. Both lattice parameter and crystal size values depend on the substrate strain character, indicating that tensile strain is maintained up to larger thicknesses of the film. The ferromagnetic order was observed in all samples, with a Curie temperature approaching, in general, the bulk value when film thickness increases. The strain imposed by the substrate was also responsible for a soft magnetic contribution, which stands out mainly in the thinner samples. The magnetism in LSCO films depends strongly on the competition between both shape and perpendicular anisotropies. The in-plane tensile strain keeps the magnetization in the film plane, whereas the compressive strain forces the easy axis to point out of the film.
